# Surgical outcomes of laser in situ keratomileusis (LASIK) in patients with stable systemic disease

**DOI:** 10.1007/s10792-024-02956-7

**Published:** 2024-02-29

**Authors:** Amr Saad, Jenny Narr, Andreas Frings, Johannes Steinberg, Toam Katz, Stephan Johannes Linke

**Affiliations:** 1 Department of Ophthalmology, Heinrich-Heine University, Duesseldorf, Germany; 2 Department of Ophthalmology, University Medical Center, Hamburg, Germany; 3 Augenheilkunde & Augenlaser Zentrum PD Dr. med. A. Frings, Nuremberg, Germany; 4 Zentrum Sehstärke, Hamburg, Germany; 5 Care-Vision, Hamburg, Germany

**Keywords:** Refractive surgery, Systemic disease, Laser in situ keratomileusis, Contraindication, Refractive outcome

## Abstract

**Purpose:**

To evaluate the surgical outcome in terms of safety, efficacy, predictability, and retreatment rate of LASIK surgery in patients with controlled systemic diseases in comparison with healthy individuals.

**Methods:**

The retrospective study included data from 1936 eyes of 976 patients with stable systemic diseases who underwent LASIK surgery between January 2016 and June 2019. The safety, efficacy, predictability of the surgery, and retreatment rate were evaluated in comparison with a control group comprising 1951 patients. The study was approved by the local ethics committee and adhered to the principles of the Declaration of Helsinki. Statistical analysis was performed using R team and the level of statistical significance was set at *p* < 0.05.

**Results:**

All treatment groups demonstrated high safety and efficacy indices after LASIK surgery. Furthermore, the study arms demonstrated comparable predictability and retreatment rates to the control group in nearly all cases. Retreatment rates were significantly higher in the rheumatoid arthritis group (*p* = 0.03), while safety indices were significantly lower in the hay fever group compared to the control group (*p* = 0.004). No intra- or postoperative sight-threatening complications were documented.

**Conclusion:**

Our findings suggest that selected patients with stable systemic conditions can safely undergo LASIK surgery and achieve comparable outcomes to healthy individuals. Further research is needed to better understand the treatment outcomes in this challenging patient population.

## Introduction

Laser-assisted in situ keratomileusis (LASIK) surgery has become an established and safe treatment for refractive errors over the past few decades [1]. However, certain systemic diseases, such as autoimmune or connective tissue diseases, are still considered as contraindications for LASIK. The United States Food and Drug Administration (FDA) and the American Academy of Ophthalmology (AAO) have established lists of contraindications, but these recommendations are largely based on limited evidence [2]. The presence of systemic diseases such as rheumatoid arthritis, systemic lupus erythematosus, Sjögren’s syndrome, or inflammatory bowel disease is considered as a relative contraindication, and therefore, patients with stable and asymptomatic conditions are still frequently excluded from LASIK surgery [3, 4]. However, studies on these challenging patient populations have shown the promising results with good refractive outcomes and high patient satisfaction [5]. Nevertheless, some research groups still advise against LASIK treatment for patients with active autoimmune diseases due to concerns about altered tissue response, slower wound healing, and increased risk of corneal complications [4, 6]. The evidence regarding contraindications for LASIK treatment is still insufficient and often relies on subjective expert opinions or limited case reports [7, 8].

In this study, we aim to investigate the safety, efficacy, and predictability of refractive outcomes after LASIK surgery in patients with controlled systemic diseases. The goal is to provide valuable insights that can facilitate therapy decisions and surgical planning for these controversial patient groups by contributing to the limited literature on this topic.

## Methods

In this retrospective study, we included data from 1936 eyes of 976 patients with stable systemic diseases who underwent LASIK surgery between January 2016 and June 2019. We defined nine different study arms including a control group, which consisted of 1951 patients and 3871 eyes and underwent also LASIK treatment during the same period. All eyes were matched based on age, gender, preoperative sphere and cylinder, treatment type, and eye side. All treatments were performed at private practices of “Care Vision Hamburg,” Germany, and in collaboration with the Ophthalmology Department of the Hospital Medical Center in Hamburg. All patient data were pseudonymized, and written consent was obtained from all patients before surgery. The study was conducted in compliance with the guidelines outlined in the Declaration of Helsinki approved from the local ethics committee (ID: 2021-1278). Additionally, the study was registered in the “German Clinical Trials Register” (trial number ID: DRKS00030317).

The inclusion criteria included that patients should be at least 18 years old, have had refractive stability for a minimum of one year, had a refractive error of at least 1.5 diopters (D), and did not have any ocular diseases affecting vision, keratoconus, pregnancy, lactation, or prior refractive surgical intervention. Additionally, patients with uncontrolled diabetes mellitus, untreated rheumatoid arthritis, active eye inflammation, or multiple systemic diseases were excluded from the study to avoid any bias in evaluation.

After obtaining a comprehensive patient history, which included medication history, all patients underwent a standardized ophthalmological examination to assess the risk of potential intra- and postoperative complications and determine any contraindications. The patients were asked about autoimmune and systemic diseases, medication, and current disease activity. All patients included in our study were in stable condition and under proper medical control of their respective diseases at the time of LASIK treatment. The ophthalmological examination included assessment of the anterior and posterior eye segments, intraocular pressure measurement, corneal pachymetry, and pupillometry. In addition, corneal topography and optical coherence tomography were performed. The preoperative refractive values, including subjective and cycloplegic spherical equivalent and corrected and uncorrected distance, intermediate, and near visual acuity were documented.

All treatments were performed under topical anesthesia following a standard protocol. First, the eye axis is marked in a sitting position. After the patient’s positioning on the laser platform and the disinfection procedure, a microkeratome is used to create a corneal flap, which is then lifted to expose the stroma for excimer laser ablation. Finally, the flap is repositioned to complete the procedure. Postoperative examinations were conducted one day, one week, one month, and three months after surgery. Patients were also instructed to use antibiotic and steroid eye drops for one week postoperatively following a standardized eye drop plan. After the first week, antibiotic and steroid eye drops were stopped, and patients were instructed to use preservative-free artificial tears for at least four weeks.

Safety of the surgery was evaluated as the percentage of eyes that lost two or more Snellen lines postoperatively, and efficacy was defined as the percentage of eyes with a visual acuity of 20/20 or better after surgery. Predictability refers to the percentage of eyes whose postoperative refraction is within ± 0.5 or ± 1 D of the target refraction. The efficacy index (EI) describes the ratio of postoperative uncorrected distance visual acuity (UDVA) to preoperative corrected distance visual acuity (CDVA), while the safety index (SI) describes the ratio of postoperative CDVA to preoperative CDVA. High values of EI and SI indicate better outcomes, with a cutoff of 0.8 for EI indicating a successful surgical outcome.

Statistical analysis was performed using R team software and statistical differences between groups were considered significant at *p* < 0.05. Normality was checked before *t*-tests were conducted. For independent groups, the unpaired Student’s *t*-test was used, while for paired groups, the paired Student’s *t*-test was used. The Fisher exact test was used to compare percentages. The odds ratio was used to measure the association between two variables and the Mann–Whitney rank test was used when the normality assumption was not met. Finally, retreatments were evaluated using Kaplan–Meier curves and the log-rank test.

## Results

In this study, we analyzed eight different patient groups, evaluating their efficacy, safety, predictability, and retreatment rates, and compared each of these outcomes with those of the control group.

### Patients with antidepressant use

**Fig. 1:**
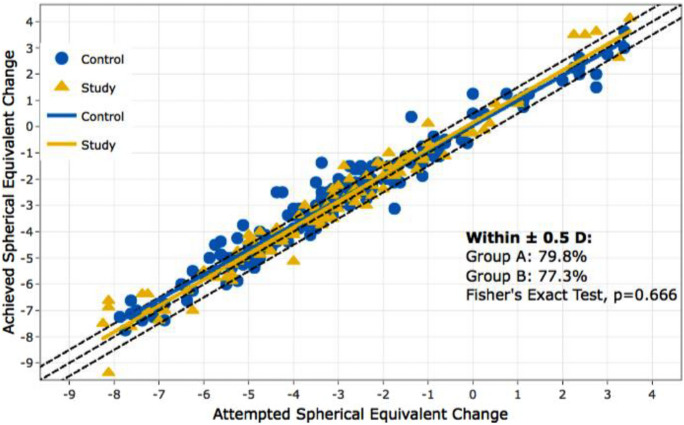
Predictability of spherical equivalent of manifest refraction in antidepressant group and control collective; Group A = control group, Group B = antidepressant group

In the first study arm, 93 patients who were taking antidepressants were included, with 91 of them undergoing bilateral LASIK treatment. The safety index for this group was 1.02 (SD = 0.13), indicating a high level of safety, with no significant difference compared to the control group. The efficacy index (EI) was 0.87 (SD = 0.21). The predictability and retreatment rates were similar between patients with antidepressant use and the control group, with 77.3% and 79.8% of patients achieved the target refractive outcome within ± 0.5 D in the antidepressant group and control group, respectively (Fig. 1).

### Patients with Bechterew’s disease (ankylosing spondylitis)

**Fig. 2:**
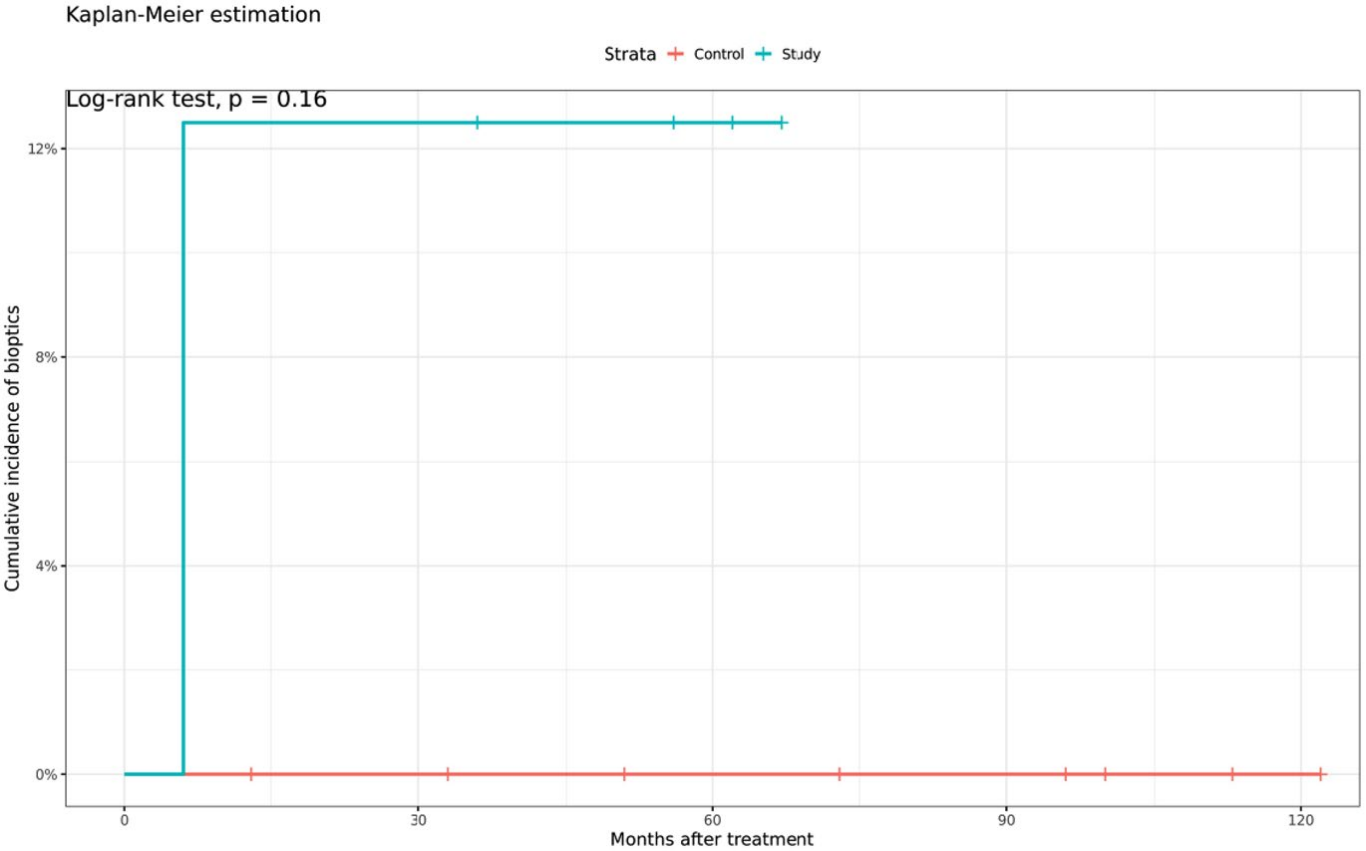
Retreatments in the group of Bechterew’s disease patients (blue) and the control group (red)

The second arm included 8 eyes of 4 patients with Bechterew’s disease. The findings indicated no significant difference in safety or efficacy compared to the group. All patients achieved a postoperative uncorrected distance visual acuity of 20/20, with no decline in vision in either group. The predictability outcomes were similar in both groups with no statistically significant difference. The Bechterew’s disease group had a higher rate of retreatments after 6 months (12.5%), but this was not statistically significant compared to the control group (0%, *p* = 0.16). (Fig. 2).

### Patients with inflammatory bowel disease (IBD)

**Fig. 3:**
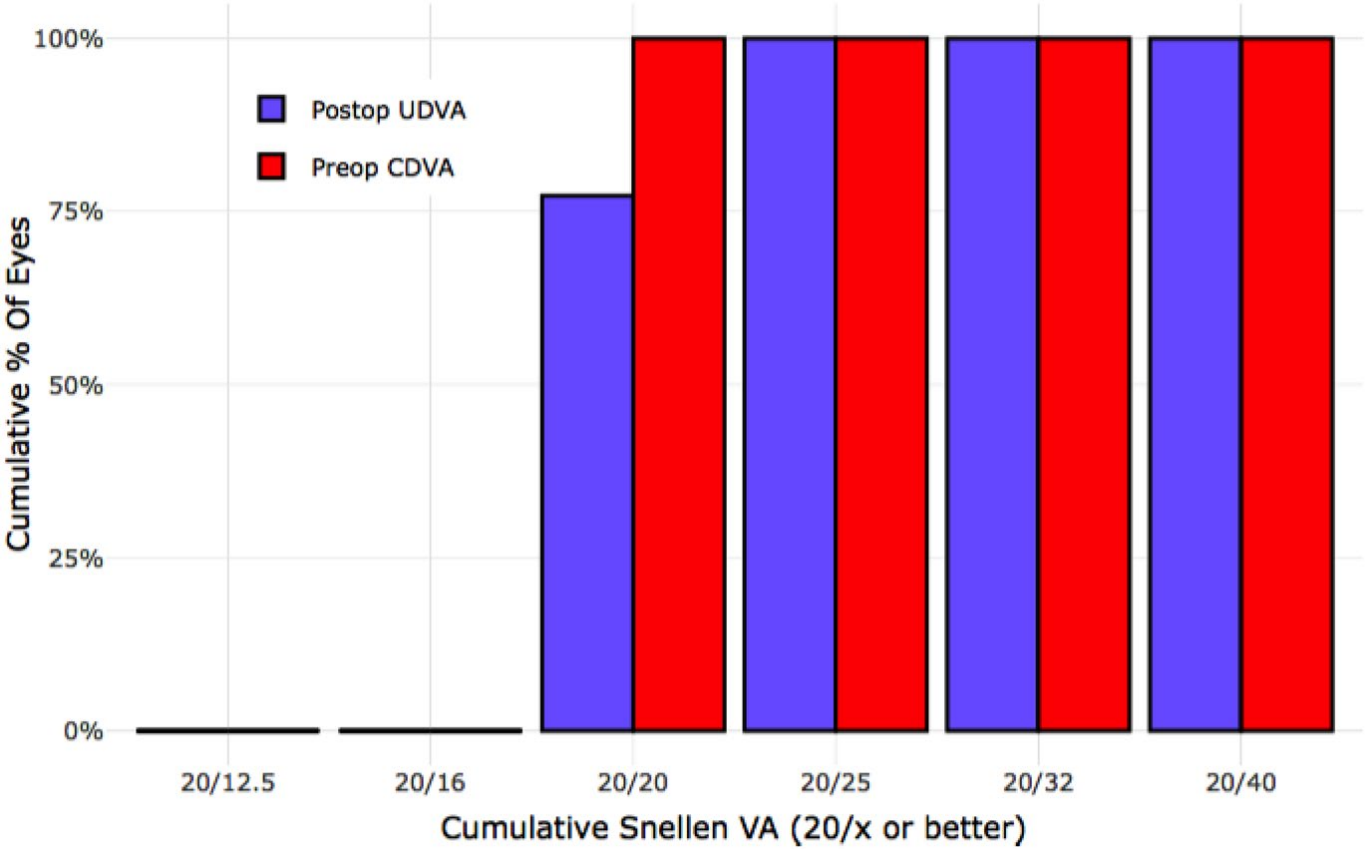
Comparison of preoperative corrected distance visual acuity (CDVA) with postoperative uncorrected distance visual acuity (UDVA) in patients with inflammatory bowel disease (IBD), illustrating the efficacy outcomes

In the third group, we included 14 patients with chronic inflammatory bowel disease (IBD) who underwent LASIK surgery on both eyes. The average age of these patients was 33 years (SD = 10). We could not find a significant difference between IBD patients and the control group in terms of safety and efficacy. The safety index was 0.97 (SD = 0.16), indicating high safety of the LASIK procedure. All IBD patients achieved a postoperative UDVA of 20/25, and 77.3% achieved a postoperative UDVA of 20/20. (Fig. 3).

Most of the IBD patients (86.4%) achieved target refraction within ± 1.0 D, which was comparable to the control group. In terms of retreatments, there was no significant difference between the patient group and the control group (*p* = 0.28).

### Patients with diabetes mellitus

**Fig. 4:**
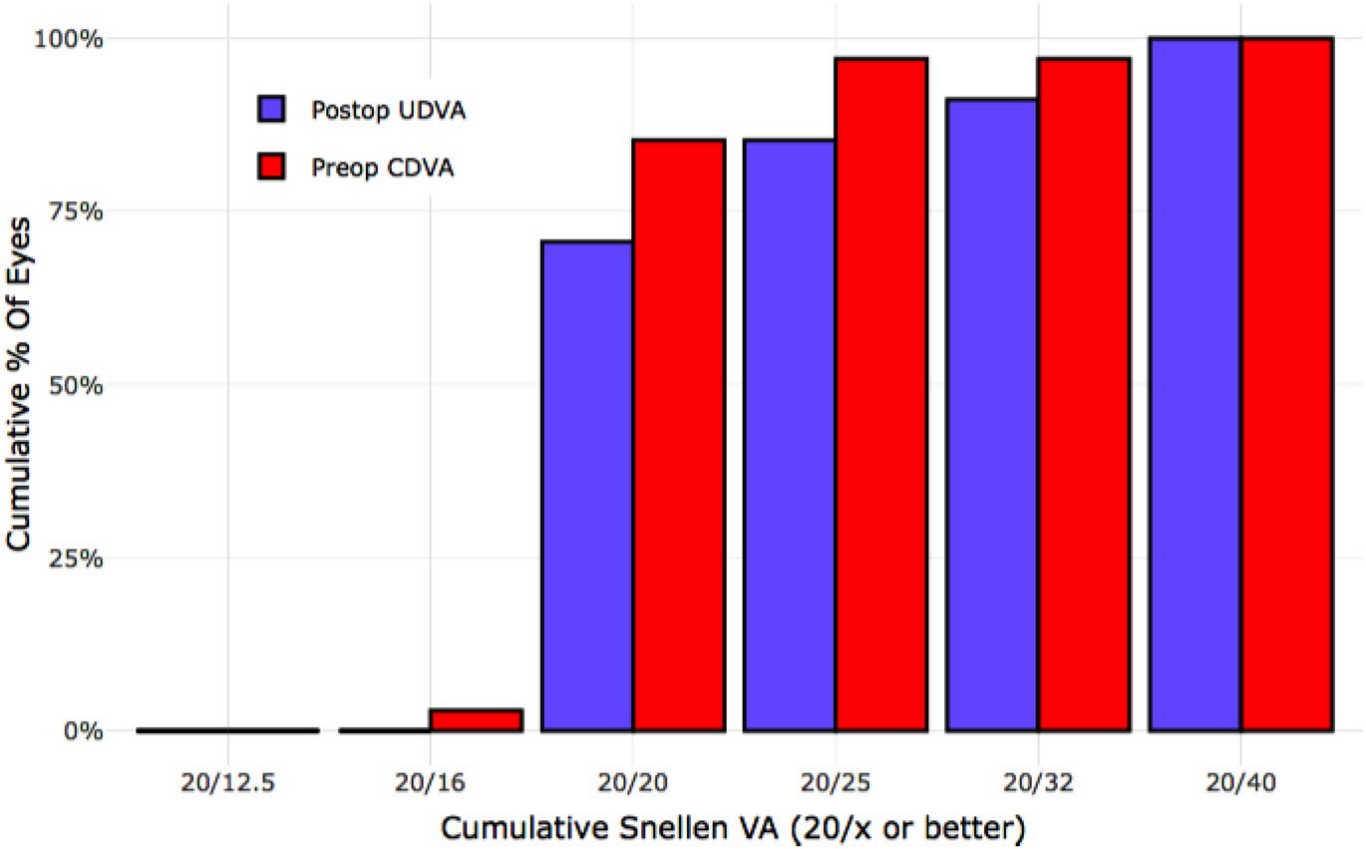
Comparison of preoperative corrected distance visual acuity (CDVA) with postoperative uncorrected distance visual acuity (UDVA) in patients with diabetes mellitus, illustrating the efficacy outcomes

This study group involved 24 diabetic patients who received LASIK surgery in both eyes. The safety results showed that 93.8% of eyes maintained the same CDVA after LASIK as before surgery, with a safety index of 1.00 (SD = 0.13). The efficacy results indicated that 91.2% of patients achieved an UDVA of 20/32 or better, while 70.6% achieved UDVA of 20/20 or better. (Fig. 4).

In both cases there were no significant differences found compared to the control group. In addition, 81.2% of the diabetes patients achieved target refractive outcome within ± 1.0 D. In terms of retreatment rates, there was no significant difference between the two groups.

### Patients with neurodermatitis

**Fig. 5:**
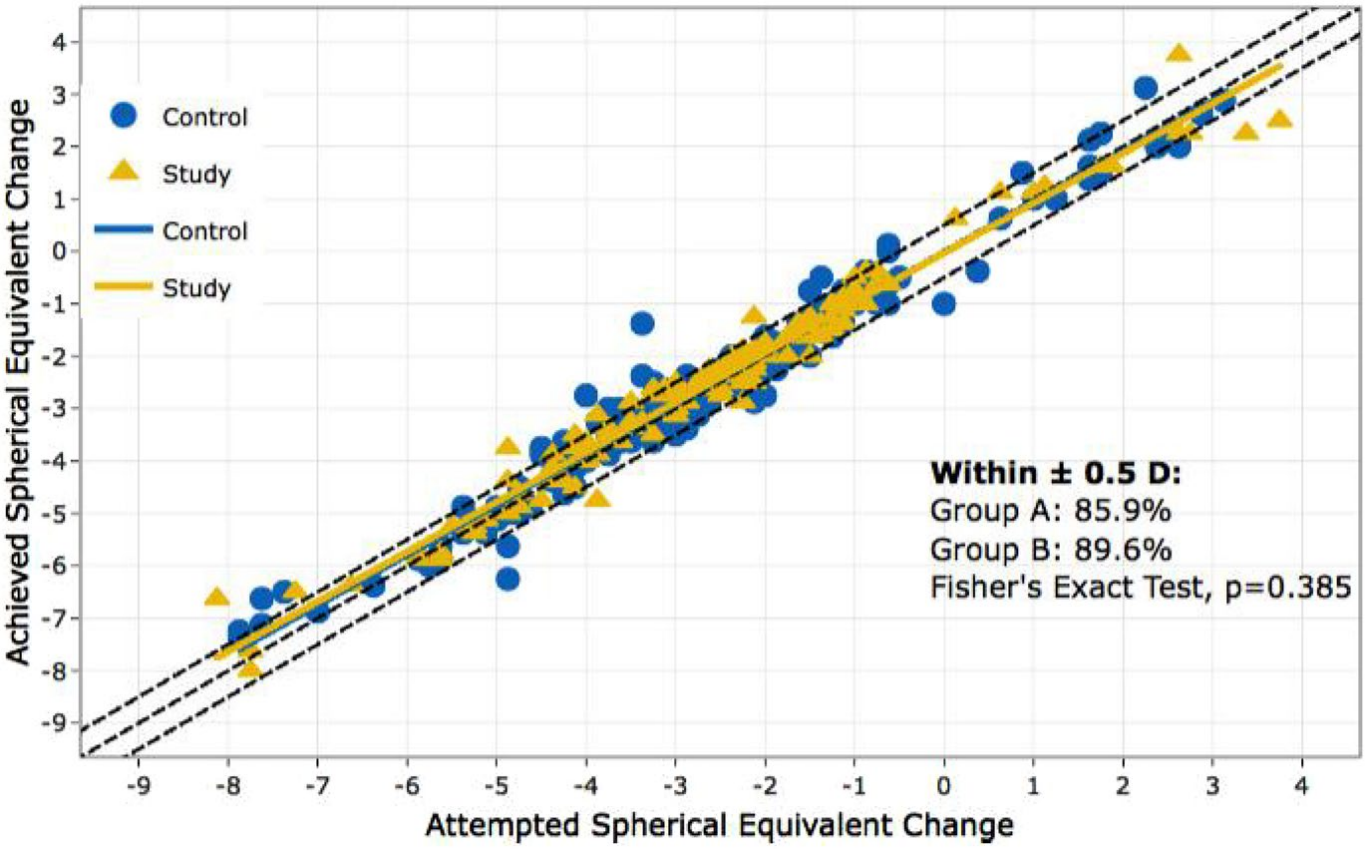
Predictability of spherical equivalent of manifest refraction in neurodermatitis group and control collective; Group A = control group, Group B = neurodermatitis group

The neurodermitis group consisted of 87 patients, with 85 of them being treated bilaterally. There were 62 female (71.3%) and 25 male (28.7%) patients in the study group. The safety index in the neurodermatitis group was 1.01 (SD = 0.13) and the efficacy of treatment was also high, with a postoperative UDVA of 20/20 achieved by 87.7% of patients. There were no statistically significant differences in safety or efficacy between the study and control groups. The predictability analysis showed that 89.6% of the patients achieved a target refractive outcome within ± 1.0 D, which was not significantly different from the control group’s rate (85.9%, *p* = 0.39). (Fig. 5).

Additionally, the retreatment rate between the two groups was not statistically significant according to the log-rank test (*p* = 0.27).

### Patients with psoriasis

**Fig. 6:**
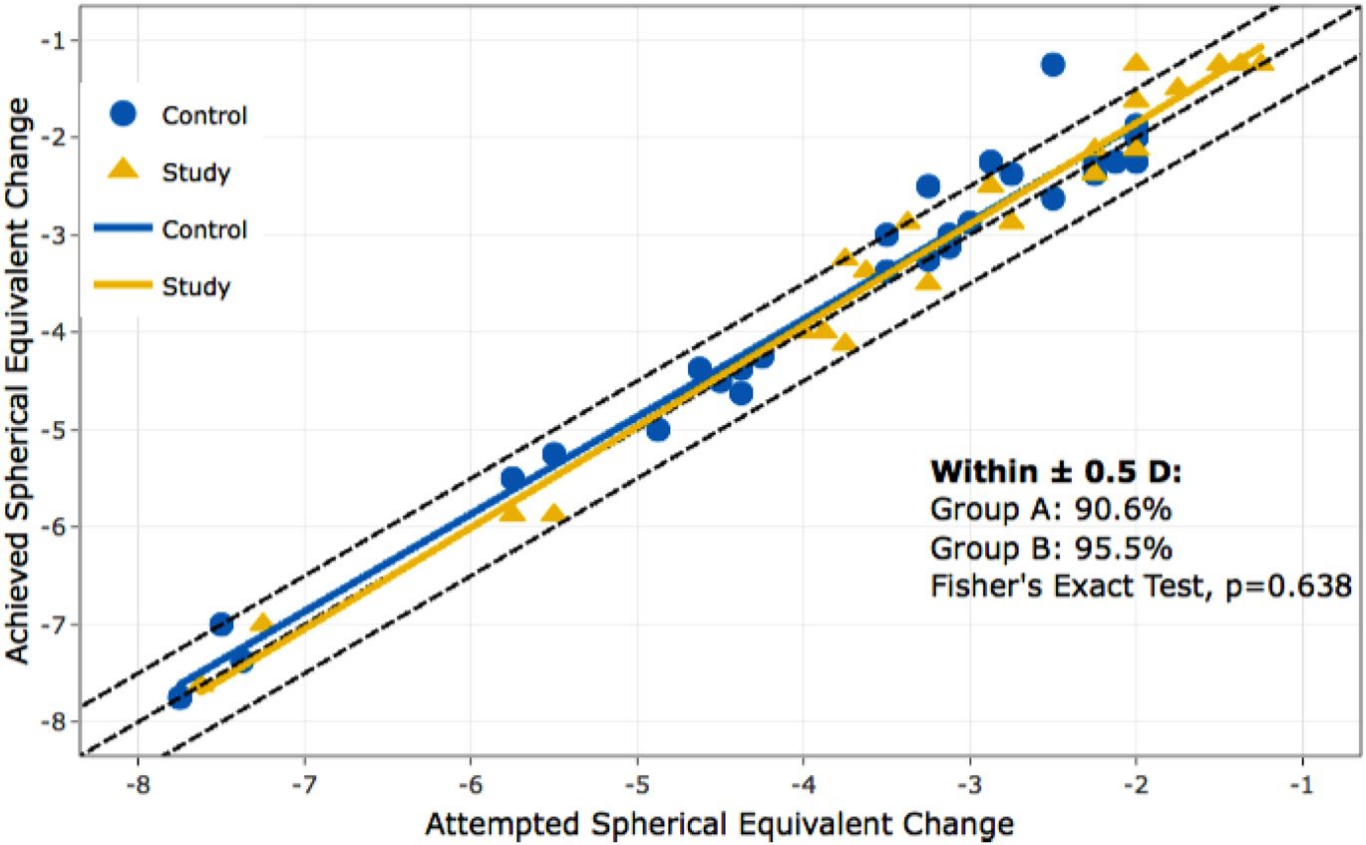
Predictability of spherical equivalent of manifest refraction in psoriasis group and control collective; Group A = control group, Group B = psoriasis group

This study arm included 14 patients with psoriasis who underwent bilateral LASIK surgery. No significant impact on the safety was found in the psoriasis patient group, with a safety index of 1.06 (SD = 0.08). Postoperatively, the psoriasis group had a high UDVA, with 81.8% of patients achieved the same UDVA as preoperatively. In terms of predictability, 95.5% of patients with psoriasis achieved the intended spherical equivalent within ± 1.0 D postoperatively, which was not statistically significantly different from the control group (90.6%, *p* = 0.64). (Fig. 6).

There was no statistically significant difference in the need for retreatment between the two groups over a follow-up period of 6 months or more (*p* = 0.91).

### Patients with rheumatoid arthritis

**Fig. 7:**
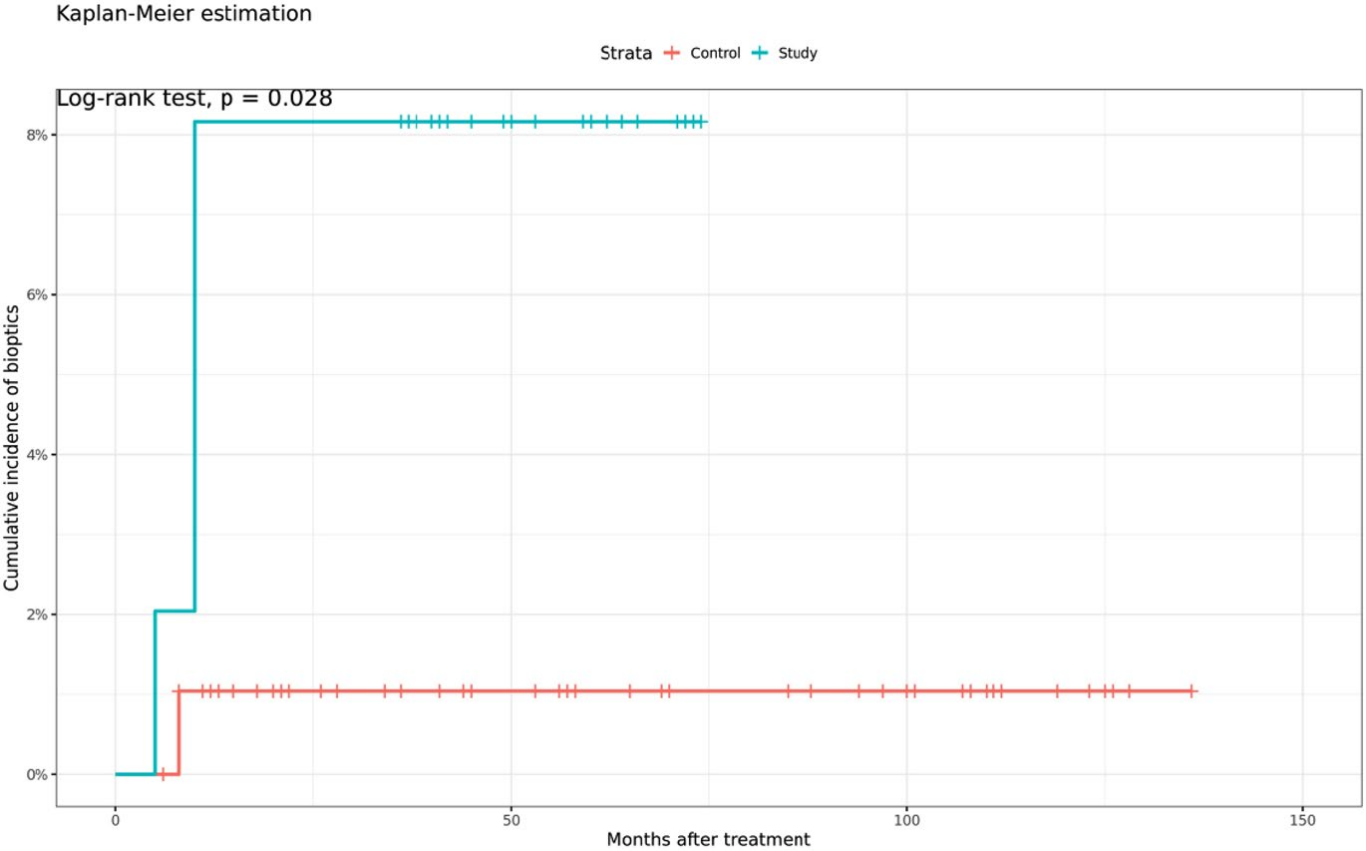
Retreatments in the group of rheumatoid arthritis patients (blue) and the control group (red)

This study group consisted of 25 patients with rheumatism, 22 of whom were female, and 3 were male. The results demonstrated a similar safety of LASIK treatments in rheumatism patients compared to the control group, although there was a slightly higher rate of a decrease in CDVA postoperatively in the patient group (*p* = 0.73). The efficacy of LASIK surgery was also not significantly different between the two groups (*p* = 0.14). In the patients group, 80.6% achieved the target refraction within ± 1.0 D and there was no statistically significant difference between the two groups (*p* = 0.57). However, there was a significant difference in retreatment rates between the rheumatoid arthritis group and the control group, with the rheumatoid arthritis group requiring retreatment more frequently (*p* = 0.03). The retreatment rate for rheumatoid arthritis patients was 2.04% within 6 months and increased to 8.16% within 12 months. (Fig. 7).

### Patients with hay fever

**Fig. 8:**
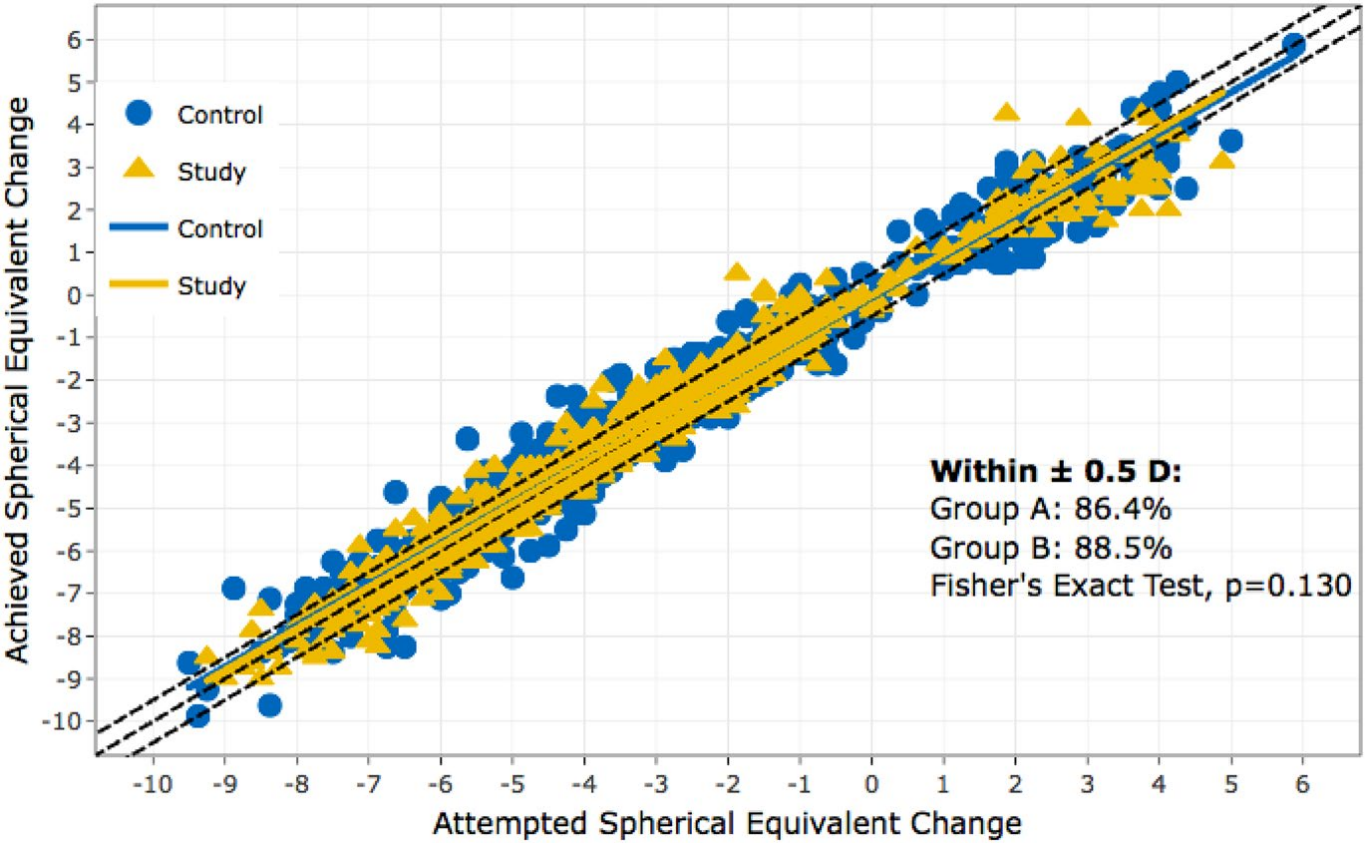
Predictability of spherical equivalent of manifest refraction in hay fever group and control collective; Group A = control group, Group B = hay fever group

The last study arm included 719 hay fever patients. The control group consisted of 1437 patients, with a similar demographic distribution. The safety index of the hay fever group was significantly lower than the SI of the control group (*p* = 0.004). The efficacy of LASIK surgery in the hay fever group was high, with most patients achieving a UDVA of 20/32 or better postoperatively (98.5%). We found that 88.4% of patients with hay fever were within the target refraction within ± 1.0 D, which is comparable to the control group. (Fig. 8).

No significant difference in retreatment rates between the two groups was noticed (*p* = 0.62).

## Discussion

The present study represents the first attempt to evaluate the refractive outcome of LASIK surgery in a large number of eyes with various systemic diseases. The data from our study showed a high safety of LASIK in patients with a history of systemic disease, including patients with IBD, diabetes, psoriasis, and rheumatologic diseases, such as Bechterew’s disease (ankylosing spondylitis). The safety results in patients with Bechterew’s disease were good and comparable to those of the healthy control group. However, caution is required in interpreting these results as the number of patients in this group was small. The safety outcomes in our rheumatics group were comparable to those reported by Cobo-Soriano et al. [3], with no loss of more than one Snellen line postoperatively.

The study found that the efficacy of LASIK In patients with IBD, diabetes, and psoriasis was comparable to other studies [3, 6]. All patients with ankylosing spondylitis achieved a 20/20 postoperative UDVA, while those taking antidepressants and with hay fever had a lower success rate potentially because of dry eye symptoms associated with their medication for the respective diseases. The EI in patients with rheumatism and ankylosing spondylitis was 0.94, which is consistent with previous literature [3, 5]. The EI in other patient populations, such as those with antidepressants, diabetes, and psoriasis, ranged from 0.87 to 0.98, all above the cutoff level of 0.8, indicating a high efficacy of LASIK in these patients. Schallhorn et al. [6] reported a 76.8% efficacy in LASIK eyes with well-controlled collagen-vascular and other immune-mediated inflammatory diseases. While they did not form subgroups for refractive outcomes, our results align with their overall findings for all patient groups.

Two studies have evaluated LASIK safety and efficacy in patients with systemic rheumatic diseases. Alio et al. [5] found that LASIK surgery is safe and effective with well-controlled diseases but emphasized the need for disease control at least three months preoperatively. Cobo-Soriano et al. [3] also found LASIK safe in patients with stable diseases, mild extraocular involvement, and well-controlled medications, emphasizing the importance of adequate disease control before refractive treatment. They reported only mild complication, such as microfolds of the flap or interface reactions, which healed with topical therapy. Despite the promising results, the authors of the study recommend excluding patients with active autoimmune diseases from LASIK and draw attention that their findings are limited to patients with controlled and stable systemic diseases.

The results of our study suggest good predictability of LASIK in all patient populations, except for those patients taking antidepressants, who showed reduced predictability. According to Cobo-Soriano et al. [3] patients with collagen-vascular disease showed a lower predictability due to undercorrection and subsequent refractive regression. However, it should be noted that the sample sizes of the different studies were different from ours and some patients were lost during the follow-up, which makes direct comparisons difficult.

The retreatment rate for patients with chronic inflammatory bowel disease and diabetes was comparable to or slightly higher than rates reported in the literature [3, 9]. Similarly, retreatment rates for patients with psoriasis, rheumatoid arthritis, neurodermatitis, and hay fever were slightly higher than those reported in previous studies [3]. Interestingly, there was no significant difference in retreatment rates between patients with depression and the control group, despite previous research suggesting that depression may lead to increased dissatisfaction with postoperative results [10].

On the one hand, refractive outcomes of the diabetic group were satisfying, which is consistent with previous studies [9]. However, it is recommended that only patients with well-controlled blood sugar levels and without systemic or ocular diabetic complications be considered for LASIK surgery. The type of diabetes did not seem to have a significant impact on the outcome of LASIK surgery [11]. On the other hand, patients with hay fever showed statistically significant worse refractive results, which could be attributed to dry eye syndrome [12]. Patients with allergies have a higher risk of developing dry eyes, and medication with antihistamines also contributes to the development of dry eyes [13]. Therefore, careful preoperative screening and postoperative follow-up are important to minimize complications. Patients should be informed of their specific risk for complications after LASIK surgery in addition to the general risks.

One limitation of our study is the short follow-up period of only three months, which may not have been sufficient to detect all possible complications. Although most complications tend to occur within the first few weeks or months [14, 15], it is possible that some complications may have arisen beyond this period. Additionally, the small size of patient groups with different systemic diseases may limit the generalizability of the study’s findings to larger populations. Nevertheless, the unique inclusion of patients with systemic diseases interested in refractive treatment still makes a valuable contribution to the literature.

This study aimed to gain further insights into the safety, efficacy, and predictability of LASIK surgery in controversial patient populations. In conclusion, we found that selected patients with certain systemic conditions, such as disease inactivity and the absence of ocular involvement, can safely undergo LASIK surgery and achieve comparable refractive outcomes to healthy individuals. We need more research and international publications on treatment outcomes after LASIK in patients with systemic diseases to increase the evidence in the treatment of this challenging patient populations.
